# Glutamate Neurocircuitry: Theoretical Underpinnings in Schizophrenia

**DOI:** 10.3389/fphar.2012.00195

**Published:** 2012-11-26

**Authors:** Thomas L. Schwartz, Shilpa Sachdeva, Stephen M. Stahl

**Affiliations:** ^1^Department of Psychiatry, State University of New York Upstate Medical UniversitySyracuse, NY, USA; ^2^Department of Psychiatry, University of California San DiegoSan Diego, CA, USA; ^3^Cambridge UniversityCambridge, UK

**Keywords:** schizophrenia, dopamine hypothesis, glutamate hypothesis, NMDA receptor

## Abstract

The Dopamine Hypothesis of Schizophrenia is actively being challenged by the NMDA Receptor Hypofunctioning Hypothesis of Schizophrenia. The latter hypothesis may actually be the starting point in neuronal pathways that ultimately modifies dopamine pathways involved in generating both positive and negative symptoms of schizophrenia postulated by the former hypothesis. The authors suggest that even this latter, NMDA receptor-based, hypothesis is likely too narrow and offer a review of typical glutamate *and* dopamine-based neurocircuitry, propose genetic vulnerabilities impacting glutamate neurocircuitry, and provide a broad interpretation of a possible etiology of schizophrenia. In conclusion, there is a brief review of potential schizophrenia treatments that rely on the etiologic theory provided in the body of the paper.

## Introduction

**Figure 1:**
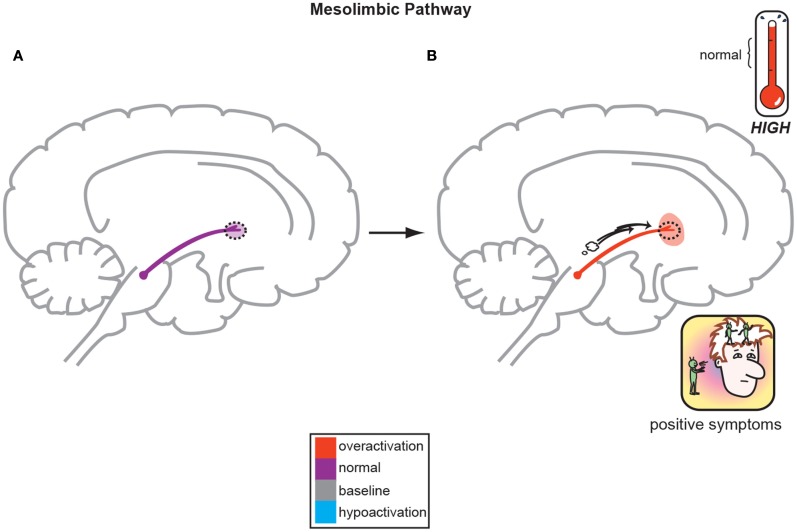
**The Dopamine Hypothesis suggests quite simply that normal, baseline mesolimbic dopamine output yields normal psychiatric functioning (A) but the positive symptoms of schizophrenia are a direct result of too much DA neuronal firing originating in the midbrain and allowing excessive DA release and activity in limbic structures (B; Stahl, 2008)**.

**Figure 2:**
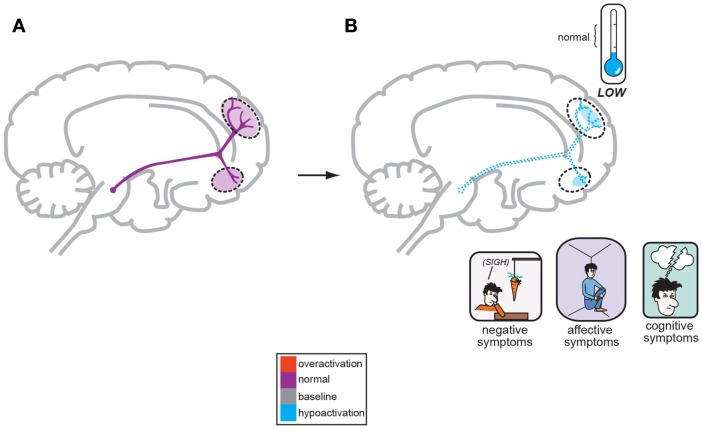
**A corollary of the original Dopamine Hypothesis suggests that normal psychiatric functioning occurs as a result of baseline or normal dopamine output reaching the frontal cortex (A), but the negative symptoms of schizophrenia are a direct result of too little DA neuronal firing originating in the midbrain and allowing poor DA release and activity the frontal cortex (B; Stahl, 2008)**.

Hypotheses for the origin of schizophrenia symptoms have likely moved further than the original “Dopamine Hypothesis” where it is postulated that overactive mesolimbic dopamine (DA) neurons cause the positive symptoms of psychosis and the corollary that underactive mesocortical DA neurons, cause the negative, cognitive, and affective symptoms of schizophrenia. For more than 30 years, this key hypothesis has dominated theories of schizophrenia and is placed prominently in every psychiatric textbook written today. This theory was reverse engineered based initially upon observations that drugs that increase DA, such as amphetamine and cocaine, can create psychotic symptoms, whereas antipsychotic drugs that decrease DA by antagonizing dopamine D2 receptors actually diminish psychotic symptoms (Meltzer and Stahl, 1976). Figure 1 displays the overactivity of mesolimbic DA circuitry as the basis for development of positive symptoms, and Figure 2 depicts the hypofunctioning of the mesocortical DA pathway which projects to the frontal cortex (Meltzer and Stahl, 1976; Stahl, 2007a). This *hypofrontality* is the proposed mediator of negative, cognitive, and affective symptoms of schizophrenia.

The Dopamine Hypothesis of Schizophrenia has been accepted as fact and antipsychotic medications have continued to be developed based upon the mechanism of antagonizing D2 receptors in hopes of lowering the firing and activity of the mesolimbic DA pathway. Clinicians from the 1950s onward were able to use the typical antipsychotics that were first developed from phenothiazine chemical structures and then utilized other chemical classes, i.e., butyrophenones, thioxanthenes, etc., as antipsychotic agents. In the 1990s, the atypical antipsychotics were developed, branded, and marketed with a dual serotonin-dopamine receptor antagonism (SDA) mechanism of action whereby they simultaneously block both D2 and serotonin-2A (5HT-2A) receptors allowing for adequate antipsychotic effectiveness while lowering the risk of extrapyramidal syndromes (EPS). This improved neuromuscular safety profile occurs as the 5HT-2A receptor antagonism allows these novel agents to be more selective at dampening mesolimbic DA activity while allowing less interference in the nigrostriatal DA pathway (Stahl, 2007a; Opler and Opler, 2012).

Interestingly, from a clinician’s point of view it is not often asked, “where did the excess dopamine activity come from?” Simplistically, a schizophrenic might have too much DA production, too little catabolism, too active or sensitive D2 receptors, etc. Schizophrenia therefore was postulated to be an illness based upon the Stress-Diathesis Model (Sadock and Sadock, 2003; Straub and Weinberger, 2006) where an individual likely has inherited one or more genes that code for abnormal proteins, and these proteins likely modify the way the mesolimbic DA pathway operates. The net result is that these abnormal proteins, i.e., receptors, enzymes, etc. likely impact upon the mesolimbic DA system making it hyperactive resulting in psychotic symptom development (Stahl, 2007a). This diathesis, or biological risk, also has to be paired with environmental stress to create enough symptoms to warrant the syndromal diagnosis of schizophrenia.

If schizophrenia developed only out of the handful of DA related genes in the human genome, then researchers should be able to create much more consistently and fully effective drugs for treating positive and negative symptoms. After 60 years of research, there are now likely safer antipsychotic medications, but they have failed to become more effective overall. Thinking outside of the box would suggest that there has to be more to the pathology of schizophrenia than just DA neurons that project from the ventral tegmental area (VTA) to the limbic structures of the brain. Perhaps, elevated mesolimbic DA activity creates one form, or type of schizophrenia? Patients with “dopamine sensitive schizophrenia” can be cured and have symptom remission on the available antipsychotic agents. Clinically, like a patient with breast cancer who undergoes genetic testing to determine if her cancer is estrogen sensitive or not, a schizophrenic could be genetically analyzed to see where his genetic vulnerability lies. If this diathesis happens to be in the DA mesolimbic system, then he statistically should respond very well to any of the marketed antipsychotics. If this schizophrenic patient is not positive for DA risk genes, then he is likely to have treatment resistant or refractory schizophrenia when treated with the available antipsychotic agents.

The oversimplification dictated by the Dopamine Hypothesis and the less than stellar clinical remission outcomes with D2 receptor antagonizing antipsychotics would suggest that either DA hyperactivity is only one part of the etiology and onset of schizophrenia, or perhaps it is the final common pathway whereby stress and multiple other neurotransmitters, receptors, neuronal pathways, etc. have to become jeopardized and converge on the mesolimbic system allowing DA hyperactivity to finally ensue. It is possible that some schizophrenia patients have normal DA pathways and their symptoms originate in other neuroanatomic structures that function primarily under the influence of non-DA neurotransmitters.

In this manner, the NMDA Receptor Hypofunction Hypothesis, has garnered much research and writing. Here, a faulty series of NMDA glutamate (GLU) receptors located on gamma aminobutyric acid (GABA) interneurons are purported to ultimately allow the generation of excessive mesolimbic DA activity outlined in the original Dopamine Hypothesis (Stahl, 2007a). This GLU variable now increases the etiologic complexity regarding development of schizophrenia symptoms as there is now interplay between GLU, GABA, and DA neurotransmitters. There is also neuroanatomic complexity in that the GLU neurons originate in the frontal cortex but descend into the limbic structures to exert their control over DA functioning. Now, to develop schizophrenia, a patient might have to inherit vulnerable genes in the DA, GLU, or even GABA-based neuronal systems. This fact would indicate that new approaches geared toward developing new treatments for schizophrenia would necessarily have to begin a divergence away from mastering D2 receptor antagonism pharmacodynamically, and move toward agents with other mechanistic properties.

This paper will now focus, not only on the NMDA Receptor Hypofunctioning Hypothesis of schizophrenia, but more globally investigate how a faulty glutamatergic system may impact neuropsychiatric function and possibly be related to the onset of positive and negative symptoms of schizophrenia.

## Neuroanatomic Primer: Glutamate, GABA, and Dopamine Circuits

**Figure 3:**
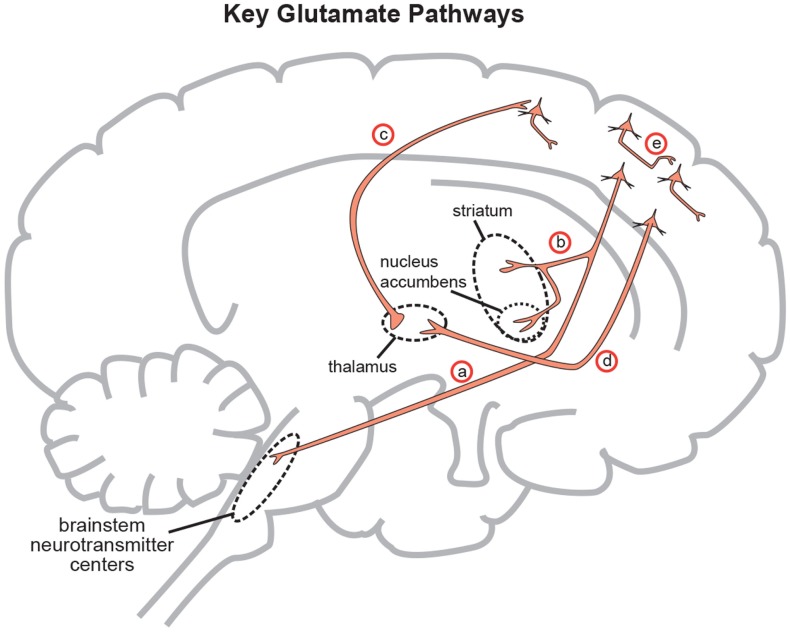
**Five glutamate pathways**. (a) The cortical brainstem glutamate projection is a descending pathway that projects from cortical pyramidal neurons in the prefrontal cortex to brainstorm neurotransmitter centers (raphe, locus coeruleus, ventral tegmental area, substantia nigra) and regulates neurotransmitter release. (b) Another descending glutamatergic pathway projects from the prefrontal cortex to the striatum (corticostriatal glutamate pathway) and to the nucleus accumbens (cortico-accumbens glutamate pathway), and constitutes the “corticostriatal” portion of cortico-striatal-thalamic loops. (c) Thalamocortical glutamate pathways are pathways that ascend from the thalamus and innervate pyramidal neurons in the cortex. (d) Corticothalamic glutamate pathways descend from the prefrontal cortex to the thalamus. (e) Intracortical pyramidal neurons can communicate with each other via the neurotransmitter glutamate. These pathways are known as cortico-cortical glutamatergic pathways. Three of the five pathways project from the frontal cortex and penetrate into deeper brain areas where they exert control over the neuroanatomic structures residing there. This paper will focus on the descending circuits associated with (a) and (b) predominantly (Stahl, 2008).

The rest of this paper and understanding of its theories necessitates understanding some basic neuronal pathways in a very simplistic form. Figure 3 depicts a series of GLU neurons that begin in the frontal cortex and connect and project in to brainstem, midbrain, and limbic areas. In this way, neurons originating in the more modern frontal neocortex may penetrate into deeper areas of the brain to exert control over midbrain centers that are primarily responsible for creating and projecting neurotransmitter activity that are ultimately responsible for drive and affective initiation. These primary GLU neurons may project further to deeper brain areas such as the amygdala and nucleus accumbens creating appropriate perceptual balance versus psychosis. This simple circuitry will be the basis for understanding the onset of negative and positive symptoms of schizophrenia.

**Figure 4:**
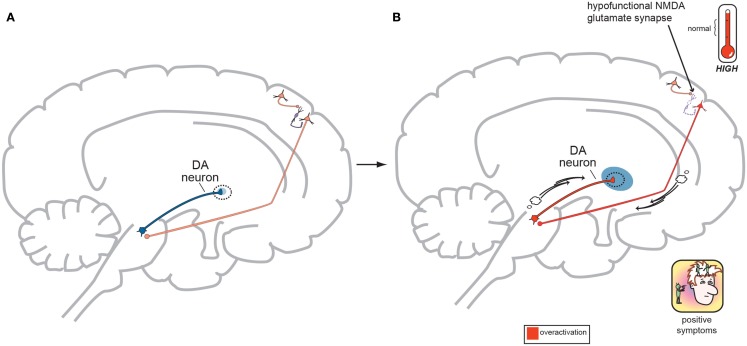
**(A)** Normal GLU-GABA-GLU-DA Neuronal Circuitry. The cortical brainstem glutamate projection starts in the frontal cortex as the top most neuron and leads ultimately to the deeper mesolimbic dopamine pathway. However, this sequence of neurons consists of several stops making a neurocircuit loop. Normally, a fully functioning primary GLU neuron (top most pyramidal neuron) fires upon a smaller GABA interneuron which next releases inhibitory GABA onto a secondary GLU pyramidal neuron (which now is the third neuron in the series from the top) causing it to lower its firing rates. This loss of GLU tone at this second GLU neuron is normal. The fourth neuron is dopaminergic and fires at a normal rate and without positive psychotic symptoms. When this circuit is optimal or controlled it avoids generation of positive schizophrenia symptoms allowing the correct amount of DA activity to occur. This creates a normal GLU-GABA-GLU-DA neurocircuit loop responsible for maintaining an appropriate, non-psychotic state. **(B)** Abnormal GLU-GABA-GLU-DA Neuronal Circuitry and the NMDA Receptor Hypofunction Hypothesis. The cortical brainstem glutamate projection in this image is compromised, or defective, in theory by less active, or suboptimal NMDA receptors. Projection starts again at the top in the frontal cortex and leads ultimately to the mesolimbic dopamine pathway again. The fully functioning primary GLU neuron fires upon a GABA interneuron which now has poorly functioning NMDA receptors situated on it. The GABA interneuron no longer fires adequately and is hypo- or under functioning. This resultant loss of GABA activity will cause the secondary GLU neuron now to abnormally increase its firing rates. This excessive GLU tone now impinges on the DA mesolimbic pathway directly stimulating firing and causing DA neuronal activity to be excessive. This now generates psychotic positive symptoms of schizophrenia. This abnormal GLU-GABA-GLU-DA neurocircuit loop now may explain the NMDA receptor hypofunction hypothesis of schizophrenia and possibly be the cause of the older Dopamine Hypothesis (Stahl, in press).

In order to focus on the development of positive psychotic symptoms, the cortical brainstem glutamate projection (Figure 3 (a)) must be examined more closely in its normal functioning state (Figure 4A) and its abnormal state (Figure 4B) as far as schizophrenia symptom development is concerned.

As outlined in Figures 4A,B below, DA neuronal activity originating in the midbrain and limbic structures does not exist in a vacuum and is likely controlled by GLU neurons in the frontal cortex to a great extent. Strong cortical primary neuronal GLU firing creates a circuit where strong GABA interneuron tone next occurs thus dampening secondary cortical GLU neuronal tone. This loss of GLU tone which arrives at the origin of the DA mesolimbic pathway can lower DA neuronal firing which is actually the normal state (homeostasis) and allows for normal psychiatric functioning. The excess DA from the Dopamine Hypothesis might actually be derived from the GLU neurocircuitry system. The NMDA Receptor Hypofunctioning Hypothesis suggests that NMDA receptors attached to the GABA interneurons situated between the primary and secondary GLU cortical neurons are to blame. These defective, insensitive NMDA receptors do not receive adequate stimulation from the primary GLU neuron thus making the GABA interneuron less effective, firing less often. This loss of GABA output onto the secondary GLU neuron allows it to fire more often, directly causing the firing of more and excessive DA (the Dopamine Hypothesis) neurons in the mesolimbic pathway resulting in psychotic symptoms. This makes the GLU-GABA-GLU-DA circuit complete, but now abnormal in functioning. The theoretical neuroanatomic circuitry involved in the Dopamine Hypothesis and the Glutamate Hypothesis has previously been briefly reviewed by one of the authors (Stahl, 2007a,b, 2008) and the genetic underpinnings of the Glutamate Hypothesis by both (Schwartz et al., 2012). Further in this paper, the authors will try to merge all accounts in order to allow the reader to think through the possible genetic origins of schizophrenia symptoms and how these phenotypic symptoms may emerge at the onset of this severe and persistent psychiatric disorder.

**Figure 5:**
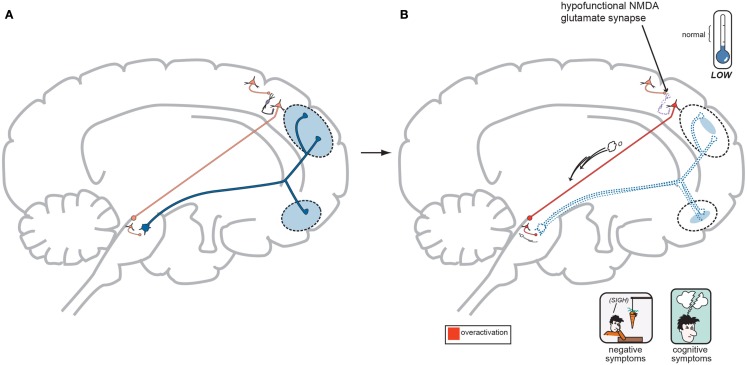
**(A)** Normal GLU-GABA-GLU-*GABA*-DA Neuronal Circuitry and the NMDA Receptor Hypofunction Hypothesis for negative symptoms. The cortical brainstem glutamate projection pathway may be involved here as well, and the circuitry involved in generating negative symptoms of schizophrenia is one step more complex. A similar relationship exists in the first three steps with a primary GLU neuron, a GABA interneuron, and a secondary GLU neuron interacting similar to Figure 4A. Next however is a synapse further down in the midbrain with yet another GABA interneuron which impinges upon DA neuronal projections that proceed back to the frontal cortex. Again, with normal psychiatric functioning, this circuit is balanced and enough DA is projected to the frontal cortex to avoid any negative symptoms. The circuit has changed from GLU-GABA-GLU-DA to one of GLU-GABA-GLU-*GABA*-DA and it has an extra step. **(B)** suggests this abnormality may cause negative symptoms. In schizophrenia, a loss of NMDA activity again may be a reasonable explanation for negative symptom development. Here, good GLU tone in the primary GLU neuron impacts dysfunctional NMDA receptors situated on the first GABA interneuron (same as noted for positive symptoms…). GABA tone is lost in the absence of GLU stimulation and the secondary GLU neuron again becomes hyperactive. Different from positive symptom generation, this secondary GLU neuron impinges upon yet another GABA interneuron and this interneuron is now stimulated by high GLU tone thus releasing much higher GABA concentrations. This increase in GABA now causes final pathway DA neurons originating in the midbrain to be inhibited and fire less. This DA mesocortical pathway is now under active and unable to supply the frontal cortex with adequate DA and *hypofrontality* and negative symptoms occur (Stahl, in press).

In regards to the negative symptoms of schizophrenia, another GLU neurocircuit must be examined (Figure 3). The cortical brainstem glutamate projection has sub-circuits that are less direct and do involve *two* series of GABA interneurons that impact upon VTA DA neurons whose purpose it is to ascend back to the dorsolateral prefrontal cortex (DLPFC) and ventromedial prefrontal cortex (VMPFC). These circuits should provide sufficient activity for alertness, concentration, emotional, and executive functioning. These abilities are often lost, or deficient in schizophrenia, and are noted as being negative symptoms (Figure 2). A loss of DA tone in this circuitry or a change in GLU tone that drives the final DA pathway may create underactivity and inefficient performance in the DLPFC/VMPFC allowing negative symptoms to develop (Figure 5). Theoretically, if a patient has inherited genes which code for abnormal proteins that impact by lowering initial GLU tone or activity in these circuits, then patients may become hypofrontal with less cortical DA activity and negative symptoms could develop as well. This type of circuit could become defective due to abnormal GLU NMDA receptors sitting on GABA interneurons, or any other impingement on the circuit that would result in final DA common pathway changes further downstream.

**Figure 6:**
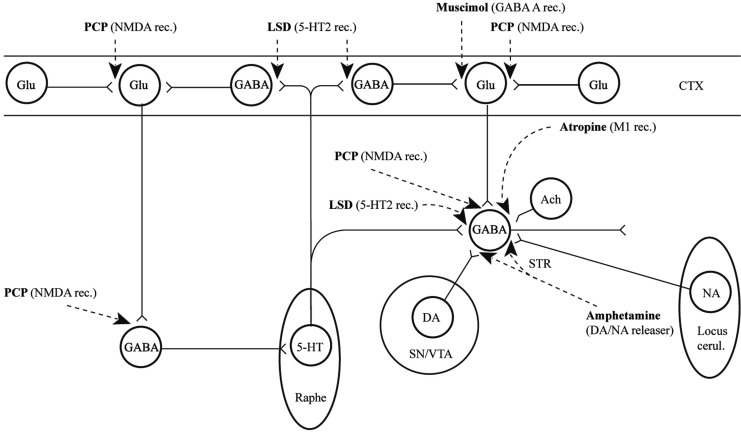
**Psychotogenic pathways vicious circle (left)**. Schematic diagram illustrating potential psychotogenic pathways and sites of action of psychotogenic and antipsychotic agents. The striatal complexes (STR, the centrally located circle) are composed of the dorsal and ventral striatum/pallidum. The striatum receives glutamatergic inputs from all parts of the cerebral cortex as well as serotonergic, dopaminergic, and noradrenergic inputs from the lower brainstem. Amphetamine and phencyclidine (PCP) are supposed to be psychotogenic by enhancing on striatal dopamine release and blocking NMDA receptors, respectively. These actions are partly located in the (limbic) striatum, partly in other sites. For example, PCP may act by blocking cortical NMDA receptors as well, e.g., in the hippocampus, as indicated in the figure, leading to reduced tone in corticostriatal glutamatergic pathways. KEY: CTX cortex, STR striatum, SN substantia nigra, VTA ventral tegmental area [With Permission Carlsson et al. (1999) and Biological Psychiatry].

**Figure 7:**
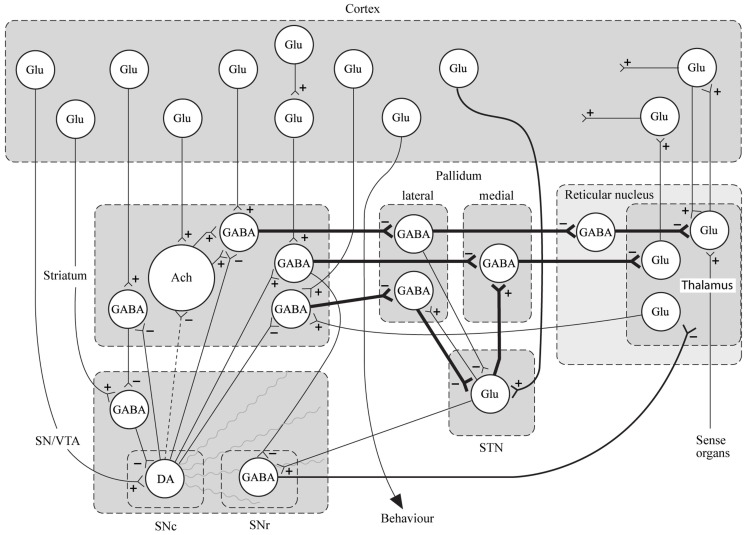
**Specific neurocircuitries of the basal ganglia**. In this figure the striato-pallido-thalamic pathways are detailed. Among these, the top and bottom pathways drawn with thick lines contain three gabaergic neurons and are referred to as “indirect” pathways. The pathway in between contains two gabaergic neurons and is referred to as “direct.” Key: SN, substantia nigra; VTA, ventral tegmental area; STN, subthalamic nucleus; Glu, glutamate; Ach, acetylcholine; DA, dopamine [With Permission Carlsson et al. (1999) and Biological Psychiatry].

In conclusion for this first section, the basic dopamine and glutamate neuropathways have been reviewed in order to alert the reader as to the current, and most accepted, individual theories behind the development of schizophrenia symptoms. They have been simplified and by using adult learning techniques (visuo-spatial processing by use of Figures, planned redundancy instead of rote memorization, mnemonics, associations between texts-figures-verbal constructs, etc.) ideally will be easier to recall and recognize factually. Later in this paper, these circuits will need to be remembered and referred to in order to understand how genetic findings and protein abnormalities might lead to the dysfunction of these neurocircuits and development of schizophrenia symptoms. For the more advanced, neuroscience or neuroanatomic reader, this simple theoretical material may be made more complex by reviewing the extensive work by Carlsson et al. (1999) and the following figures may represent the greater complexity of the neuroanatomic basis of the dopaminergic and glutamatergic hypotheses of schizophrenia (see Figures 6 and 7).

## Genetics Primer for the Glutamate Pathways Involved in Schizophrenia

Accepting that the Dopamine Hypothesis is the final common pathway to schizophrenia symptom development and the complimentary concept that under functioning NMDA receptors or malfunctioning glutamate circuitry is to blame for the ultimate schizophrenia DA abnormalities and symptoms, it now makes sense to better explore how, or why, the GLU system might become dysfunctional in the first place. Again, patients inherit genes that code for proteins. If the gene is mutated or abnormal, then an abnormal protein is created and mass produced. If any of these proteins affect, or are in the vicinity of the GLU pathways, (Figure 3) then the GLU system may falter and change its overall tone. The downstream effect may be to increase DA in the mesolimbic system creating positive symptoms or to lower its activity in the mesocortical symptom allowing for negative symptoms to occur (Stahl, 2007a, 2008).

The NMDA receptor itself is ubiquitous in the CNS and is a key factor in promoting glutamatergic neuronal activity throughout. It is comprised of several small receptor subunits, any of which, if altered by genetic mutation, might alter NMDA activity leading to schizophrenia symptoms. As outlined above, any disruption, making NMDA receptors less active may ultimately account for the genesis of both positive and negative symptoms of schizophrenia (Stahl, 2007b).

NMDA receptors have a well defined three dimensional structure, and they form heterotetramers between two NR1 and two NR2 type subunits. This forms a pore at the center which is a calcium influx channel (Furukawa et al., 2005) that allows neuronal depolarization and activation. NR3 A and B subunits have also been discovered and actually cause NMDA receptors to become less active or insensitive (Sobolevsky et al., 2002). In theory, genetic mutations coding for the protein structure of NR1 or 2 subunits, if making them less sensitive or hypoactive or those making the NR3 A or B subunits more sensitive or more active, might actually dampen NMDA receptor channel opening, allowing less calcium influx. This hyperpolarizing loss of activity might impinge on the GLU-GABA-GLU or GLU-GABA-GLU-GABA circuits outlined previously. The dysfunctional NMDA receptors, sitting on GABA interneurons in this neurocircuitry may allow for dysfunctional downstream DA activity in final common pathways to create positive or negative symptoms respectively.

The gene, *GRIN1*, codes for the NR1 NMDA receptor subunit and *GRIN1* gene abnormalities are a key research interest as a susceptibility gene for schizophrenia. Mice lacking NR1 subunits (gene knock-out mice) exhibit signs that mimic human schizophrenia symptoms (Mohn et al., 1999; Halene et al., 2009). NMDA receptors most often consist of an NR1 subunit (coded by the *GRIN1* gene) plus one of the four types of NR2 subunits (*GRIN2A*, *GRIN2B*, *GRIN2C*, and *GRIN2D* genes). The two subunits of an NMDA receptor may allow for varying functions, as the NR1 subunit possesses the characteristic ion channel properties but the specificity regarding how the NMDA receptor will ultimately function and react is derived from the NR2 subunits housed in the receptor complex. In this manner, NMDA receptors with different functional capacities may be situated in the brain in order to make neurocircuits more, or less active (Moriyoshi et al., 1991; Hollmann and Heinemann, 1994; Nakanishi and Masu, 1994). Summarizing a lengthy evidence base regarding NR1 subunit genetics, there are mixed outcomes showing possible NR1 mutations leading to, or protecting against, the development of schizophrenia. Low NR1 expression due to *GRIN1* mutations likely affords low NMDA activity. By re-reviewing Figure 4, notice that these mutations may provide a low level of NMDA activity, and if this occurs at GABA interneurons in the GLU-GABA-GLU circuit, then DA excess and psychotic symptoms may occur if the mutated NMDA receptors reside in the cortical brainstem glutamate projection pathway. Likewise negative symptoms may occur if the cortical brainstem glutamate projections are compromised in the GLU-GABA-GLU-GABA circuit (Figure 5).

*GRIN2A* is the gene that codes for the NR2A subunit. These often appear preferentially in the frontal cortex and increase in the teen years (Watanabe et al., 1993; Mohrmann et al., 2000), uncanny in a clinical sense, as this age often heralds the onset of schizophrenia. Mice lacking the equivalent of the *GRIN2A* gene cause abnormal mouse behaviors similar to those observed in animal model schizophrenia (Miyamoto et al., 2001). Literature reviews support a stronger evidence base regarding NMDA receptor hypofunctioning related to abnormal *GRIN2A* genetic vulnerabilities when compared to *GRIN1A*. NR2B subunit genetic data suggests mixed results in regards to its conveying risk for schizophrenia. Stronger data may actually support an interaction between these genes, i.e., *GRIN1A* plus *GRIN2B*, and the interactions between abnormal genes and their resultant proteins may actually convey greater risk for schizophrenia than any single gene alone (Williams et al., 2002; Loftis and Janowsky, 2003; Qin et al., 2005; Tang et al., 2006).

There also exist genes that code for proteins, outside of the NMDA receptor complex that may be involved in the etiology of schizophrenia. These proteins may either directly impact the functioning of the NMDA receptor itself, making it hypoactive in nature, or these proteins may cause a dampening of GLU neuronal activity with the same net effect of allowing terminal DA abnormalities in the GLU-GABA-GLU-DA or GLU-GABA-GLU-GABA-DA circuit to cause positive and negative symptoms. For example, a calcineurin gene codes for an NMDA catalytic subunit and rodents missing this gene will have lower (hypofunctioning) NMDA receptor activity and exhibit psychotic model symptoms (Gerber et al., 2003; Miyakawa et al., 2003). A mutated neuregulin-1 (*NRG1*) gene’s abnormal protein may also contribute to poor NMDA functioning in rodent and human models (Fischbach and Rosen, 1997; Ozaki et al., 1997; Stefansson et al., 2002, 2003; Hahn et al., 2006). Neuregulin-1 codes for a protein that is required for normal GLU neuron dendritic spine formation. This allows for normal and efficient GLU neurotransmission and synapse formation in the GLU circuits discussed in Figure 3. Poorly functioning neuregulin-1 then would lead to diminished GLU neurotransmission regardless of NMDA receptor capabilities and secondary DA excesses through the GLU-GABA-GLU-DA circuitry yielding positive schizophrenia symptoms.

The NMDA receptor is not the only GLU receptor in the CNS. The non-NMDA ionotropic glutamate receptor kainate-3 gene (*GRIK3*) codes for glutamate kainite receptors and certain mutations here may pose a possible risk for schizophrenia (Begni et al., 2002). Metabotropic (non-NMDA, non-ligand gated channel) glutamate receptors also exist and their proteins are encoded by the genes *GRM2*, *GRM5*, *GRM7*, and *GRM8*. Of these, the GRM5 mutation has revealed the most significant results (EmDevon et al., 2001).

Gene *G72* codes for a protein that interacts with d-amino acid oxidase enzyme which oxidizes d-serine that usually activates NMDA receptors making them more efficient. *G72* mutations might allow for underactive NMDA receptor activity by lowering this enzymatic activity (Chumakov et al., 2002). *DTNBP1* (dystrobrevin binding protein 1) codes for dysbindin proteins that are noted to be reduced in the hippocampi and likely frontal lobes of schizophrenia patients. Altered expression of dysbindin likely lowers the activity of lysosome related organelle complexes (BLOC-1) which may lead to abnormal protein distributions in schizophrenia brains. Therefore, dysbindin may influence and cause excitotoxic GLU release increasing neuronal cell death. A reduction in dysbindin activity due to mutations might lead to a hypoactive glutamatergic system as GLU neurons die and are pruned allowing a global GLU hypofunctioning to occur, again regardless of NMDA status (McClintock et al., 2003; Talbot et al., 2004). *Akt1* genes code for protein kinase B and are needed for adequate dysbindin functioning. Mutations of Akt1 may subsequently cause poor dysbindin activity and ultimately less glutamatergic tone (Emamian et al., 2004). *RGS4* coded proteins are decreased in the prefrontal cortex of schizophrenic patients and mutations may alter G protein mediated signaling via DA, metabotropic GLU, and muscarinic cholinergic receptors. Again, a mutation here does not directly impact NMDA receptors, but would diminish metabotropic GLU receptor activity. This metabotropic input loss, next lowers NMDA receptor activity and lowers GLU tone with the final common pathway being exhibited by DA pathway abnormalities (Chen et al., 2004; Morris et al., 2004). Serine racemase (SRR) enzyme converts l-serine to d-serine, and the latter is a co-agonist of the glycine site of NMDA receptors. NMDA receptors require glycine activity to become fully active, depolarize, and provide optimal neuronal firing. d-Serine also facilitates NMDA firing in a similar manner. Mutations allowing low SRR activity would lower the co-agonist properties, thus lowering the ability of NMDA receptors to be appropriately active. This is another level of potential GLU circuitry hypofunctioning (Morita et al., 2007).

Finally, in the CNS, nitric oxide (NO) has been shown to influence the release of neurotransmitters, learning, memory, and neurodevelopment (Maia de Oliveira et al., 2011) by facilitating neuronal maturation and synaptogenesis. Disturbances in NO release could interfere with both the maturation of cortical neurons and the formation of viable synaptic connections, in accordance with the neurodevelopmental hypothesis of schizophrenia and even during normal synaptic pruning in adolescence and synaptic plasticity into adulthood. Genetically, the promoter region of NO synthase-I (encoded by the gene *NOS1*), has been studied in regards to cortical glutamate transmission and been associated with schizophrenia symptoms (Reif et al., 2011). Typically, NOS will generate NO gas, a neurotransmitter, that ultimately functions to enhance GABA interneuron tone and thus lower secondary glutamate neuronal activity. This may protect frontal cortical neurons from excitotoxic destruction. Schizophrenics with specific NOS1 risk alleles (rs41279104 AA/AG) when studied by functional imaging showed slower dorsolateral cortical functioning consistent with hypofrontal negative symptoms. This may occur as this risk gene lowers NOS1 production and secondarily NO levels in the cortex. This causes GABA interneurons to lose tonic firing, lowering of inhibition upon secondary glutamate neurons that may now promote excessive glutamate activity, neuronal destruction, and hypofrontality. Deutsch et al. (1997) conducted a small, open label study using methylene blue to alter the NO pathway and found modest improvements in test subjects with schizophrenia possibly confirming this theoretical model translationally.

All of these aforementioned genes and their protein products do not directly affect NMDA receptor functioning by altering NMDA subunits, but may lower GLU tone in GLU neurons allowing the same downstream effects and schizophrenia symptoms as if the NMDA receptors themselves were hypofunctioning. The above, gene-laden paragraph should lead the reader to the conclusion that there is not one gene that causes schizophrenia but possibly several that may impact the GLU neurocircuitry and if many of these genes are inherited then the patient now has an increased risk to develop schizophrenia. This further alerts that reader that the Dopamine Hypothesis was too simple. It is clear that the D2 receptor antagonists, first and second generation antipsychotics, are modest at best at reducing positive symptoms and very poor at lowering negative symptoms. Schizophrenia development is likely driven by neurodevelopmental, neurodegenerative, and functional neurotransmission abnormalities making the etiology of schizophrenia more complex than the dopamine hypothesis even when it is augmented and elaborated by way of the glutamate hypothesis.

In summary, the final common pathway of schizophrenia symptom development embodies the Dopamine Hypothesis where too much limbic DA allows for positive symptoms and too little frontocortical DA allows for negative symptoms. This is likely the tip of the iceberg in that this effect is superficial but may not fully describe or explain what is beneath. The NMDA Receptor Hypofunctioning Hypothesis looks a bit deeper and suggests that faulty NMDA receptors upstream from the DA pathways are the actual cause of the Dopamine Hypothesis. Weak NMDA receptors allow DA excesses to occur in some neurocircuits and deficiencies in others, thus promoting schizophrenia symptoms. Outside of the basic education of these two complimentary explanations regarding the possible etiology of schizophrenia, this paper sought to increase the acceptance of the idea that the NMDA receptors may be key in their own hypothesis, but more globally, any genetic mutation coding for proteins that might impact and cause hypofunctioning anywhere along certain GLU pathways and neurocircuitry may have the same effect as inheriting hypofunctioning NMDA receptors alone. This corollary to the NMDA receptor hypothesis then would suggest that poor glutamatergic tone derived from any source may next lead to the final common pathway of the Dopamine Hypothesis. The next, and final section of this paper will utilize this basic science knowledge and translationally review potential new schizophrenia treatments.

## Future Treatments

### Direct acting NMDA receptor agonists

These agents are analogs for endogenous glycine. Using these to increase synaptic concentration of co-agonists for the NMDA receptor should increase NMDA receptor activity. The net effect is greater glutamatergic tone which ideally rectifies the final common pathway of DA neurocircuitry dysfunction. d-Cycloserine is one of the most common agents studied so far and results are equivocal at best. More recently, one of the largest scale, multicenter studies (CONSIST) showed that neither glycine nor d-cycloserine separated from placebo in a randomized trial design in schizophrenia patients suffering from negative symptomatology (Buchanan et al., 2007). Meta-analytically, Tsai and Lin (2010) attempted to evaluate glutamate-based treatments for schizophrenia and analyzed 26 studies meeting descriptive stringent trial design criteria and suggest that GLU manipulating agents can be effective for both positive and negative symptoms. The most robust effect size was noted for treating depressive affective symptoms, negative symptoms, and finally positive symptoms. Full NMDA receptor agonists, glycine and d-serine, appeared to be most effective in these clinical symptom areas but partial agonist, d-cycloserine, was not. All agents were found to be well tolerated.

### Glycine transporter 1 inhibitors

Glycine transporters allow reuptake of glycine and when removed from the synapse, this co-agonist cannot facilitate further NMDA activity. Similar to SSRI for depression, blocking these transporters allows endogenous glycine to increase in synaptic concentration fostering greater NMDA activity to overcome the proposed NMDA receptor hypofunctioning postulate. Sarcosine is an experimental agent with this property. When used as adjunctive therapy with an approved antipsychotic agent, sarcosine has been shown to separate from placebo and has been validated in a few studies (Tsai et al., 2004; Lane et al., 2010). These agents appear to directly improve GLU tone and may have the ability to improve schizophrenia in a manner supporting the NMDA Receptor Hypofunctioning Hypothesis.

### Kynurenine pathway agents

In the CNS, tryptophan is metabolized and degraded into several metabolites that may inhibit the glutamate neurocircuitry at the level of the NMDA receptor. Specific metabolites of tryptophan in this pathway include: quinolinic acid which is an excitotoxic NMDA receptor agonist, 3-hydroxykynurenine which is a free-radical generator, and kynurenic add (KYNA) which is an antagonist at glutamate receptors. All of these might impair glutamate neurocircuitry and allow for schizophrenia symptoms to develop. Findings in schizophrenia show elevated KYNA which specifically blocks the glycine site on NMDA receptors (Erhardt et al., 2009) and may be more involved in the generation of negative symptoms (cognition, executive dysfunction). Interestingly, KYNA levels increase after infection. Neurodevelopmental theories of schizophrenia postulate that infections *in utero* or infancy may lend risk to developing schizophrenia. Prospective glycine agonists noted above may be useful if a schizophrenic could be identified as having excesses in KYNA, as competition at the NMDA receptor glycine site may be improved. Translationally, KAT II (Kynurenine aminotransferase: the enzyme responsible for converting tryptophan into KYNA), could be blocked or antagonized in order to lower KYNA levels and improve NMDA functioning. COX-2 inhibitors (anti-inflammatory agents) could be the initial or prototypical agents mechanistically as they lower KYNA levels.

### mGluR 2/3 presynaptic receptor agonists

In some GLU pathways, *secondary* downstream GLU neurons are actually hyperactive with increased tone. This situation is noted in the NMDA Receptor Hypofunction Hypothesis when the primary GLU neuron is active, but its GABA interneuron has ineffective NMDA receptors and the GABA interneuron will not fire and appropriately inhibit a secondary GLU neuron effectively. This secondary GLU neuron is hyperactive and likely allows DA abnormalities consistent with the Dopamine Hypothesis. Perfectly situated, mGluR 2/3 receptors are typically autoreceptors used to diminish GLU tone. Here, in this specific situation, increasing their tone with an agonist drug would seek to lower the abnormally high GLU firing in these secondary GLU neurons. LY2140023 is an experimental compound that is an mGluR 2/3 receptor agonist and was recently studied in schizophrenia in a Phase 2 trial comparing several dose strengths versus an active antipsychotic comparator, olanzapine, and placebo. Neither the experimental drug, nor the approved atypical antipsychotic separated from placebo making interpretation of results negative. The placebo rate was quite high for a schizophrenia study and four subjects suffered seizure-like activity (Kinon et al., 2011). A previous, smaller study versus placebo did show effectiveness in lowering positive and negative symptoms (Patil et al., 2007).

### Neuroprotective agents

Minocycline, an antibiotic, has gained some evidence base in the literature regarding refractory schizophrenia treatment as it may treat negative symptoms. Outside of its antibiotic effects, it is felt to inhibit microglial activation, decrease NO synthase induced apoptosis of neurons, and possibly positively modulate the GluR1 subunits of AMPA glutamate receptors. AMPA receptors were discussed only briefly above, but their dysfunction may also lead to schizophrenia symptoms (Kiss et al., 2011). Glutamate neurocircuitry disruption may occur in the thalamus, more so than the cortex where dysfunctional AMPA receptors reside. AMPA modulators have initial data in human schizophrenia trials suggesting improved long term potentiation rates in hippocampal structures where memory and cognition findings improved secondarily (Wezenberg et al., 2007). Minocycline may have its mechanism of action locally in these non-cortical CNS areas. Therefore, minocycline does not have a direct action on NMDA glutamate receptors, but may influence AMPA receptors that secondarily influence NMDA receptors and improve their activity levels theoretically in schizophrenia (Chaves et al., 2009).

*N*-acetyl cysteine (NAC) as a prescription is used as a mucolytic agent in infants for respiratory distress but is gaining increased research data and clinical use as a psychotropic. Mechanistically, NAC increases CNS cysteine levels that regulate neuronal intra- and extracellular exchange of glutamate through a shared transporter with glutamate. These transporters are mostly located on glial cells where cysteine is taken up and glutamate is released into the extracellular space. Increased free glutamate next may be able to increase the firing of inhibitory metabotropic glutamate receptors resting on glutamate neurons effectively lowering the net, overall release of glutamate (Dean et al., 2011). This may lower NMDA excitotoxicity and preserve balance between the dopamine and glutamate neuropathways. Similar to minocycline, negative schizophrenia symptoms appear to preferentially improve in regards to initial studies’ findings.

## Conclusion

Clinicians have treated schizophrenia since the 1950s assuming that DA excess in mesolimbic pathways was the cause of positive symptoms and DA deficiencies in the mesocortical pathways allowed for negative symptoms to develop. This Dopamine Hypothesis likely holds true for certain patients suffering from schizophrenia as perhaps these individuals inherited genes for DA receptors, reuptake pumps, metabolic enzymes, etc. that afforded them hyperactivity or hypoactivity in these pathways causing their schizophrenia to develop. If these abnormalities were the only cause or etiology for schizophrenia then currently available antipsychotic agents should be remarkably more effective than they currently are. Educators in psychopharmacology have likely oversimplified their ideas surrounding the pathophysiology of schizophrenia by focusing only on DA mechanisms of illness. The next most robust hypothesis is that discussed in detail above, where hypofunctioning glutamate NMDA receptors are to blame ultimately for DA activity variances which ultimately cause DA based positive or negative symptoms. The authors attempt to convey that focusing only on NMDA receptor hypotheses is also likely too simple. In theory, any genetic mutation that causes a change in functioning of certain GLU neuroanatomic pathways may achieve the same effect as having ineffective NMDA receptors in that abnormal downstream DA activity can occur at several different neuroanatomic sites causing schizophrenia symptoms to develop. Over the next several years, drug compounds focusing more on GLU systems may yield helpful treatments for schizophrenia patients.

## Conflict of Interest Statement

Drs. Schwartz and Sachdeva have no known conflicts of interest regarding any products outlined in this paper. Dr. Stahl has served as a Consultant to Abbott, Advent, Alkermes, Arena, Astra Zeneca, BioMarin, Boehringer Ingelheim, Bristol Myers Squibb, Cypress Bioscience, Dainippon Sumitomo, Eli Lilly, Forest, Genomind, Janssen, Jazz, LaboPharm, Lundbeck, Merck, Neuronetics, Novartis, ONO, Orexigen, Otsuka, PamLabs, PGxHealth, Pfizer, Rexahn, Royalty Pharma, Schering Plough, Servier, Shire, Valeant, and Vivus. He has served on speakers bureaus for Merck, PamLabs, Dainippon Sumitomo/Sepracor/Sunovion, Eli Lilly, and has received research and/or grant support from Astra Zeneca, Biomarin, Dainippon Sumitomo/Sepracor/Sunovion, Eli Lilly, Forest, Genomind, Merck/Schering Plough, PamLabs, Pfizer, PGxHealth, Servier, Shire, Torrent, and Trovis.
